# The Role of Self-Transcendence and Cognitive Processes in the Response Expectancy Effect

**DOI:** 10.5334/pb.364

**Published:** 2017-06-26

**Authors:** Megan Fresson, Benoit Dardenne, Marie Geurten, Laury Anzaldi, Thierry Meulemans

**Affiliations:** 1 Psychology and Neuroscience of Cognition (Research Unit), University of Liège, Liège, BE

**Keywords:** Response expectancy, Placebo, Nocebo, Neuropsychology, Self-transcendence, Monitoring

## Abstract

Neuropsychological assessment is known to be influenced by expectancy effects, which can either enhance (placebo) or diminish (nocebo) cognitive performance. Research suggests that the response expectancy effect is influenced by various individual and situational factors and that the placebo effect results in an increase in monitoring processes as measured indirectly. However, the impact on monitoring processes has not yet been studied by direct measures such as Judgement Of Learning (JOL). This study aimed to investigate the response expectancy effect on various neuropsychological tasks, including a task that directly assesses monitoring capacities (JOL). In addition to determining which cognitive functions are influenced by the expectancy effect, this study examined the moderating role of the self-transcendence dimension of personality. Eighty healthy subjects were exposed to three bogus conditions presented as allegedly having a positive, negative, or no impact on cognitive capacities. Then they completed, in random order, three blocks of tasks (executive, attentional, and memory), one in each condition. Results showed an effect of negative instructions on flexibility (poorer performance) and memory (better performance) scores. Furthermore, positive instructions led to better explicit monitoring capacities (JOL) than the neutral condition. These effects were moderated by self-transcendence, as only participants with moderate or high self-transcendence exhibited these effects. Overall, our results showed that the response expectancy effect emerges from a combination of individual and cognitive factors.

## Introduction

Neuropsychological performance is generally not a perfect reflection of the individual’s cognitive potential. Indeed, neuropsychological test performance is influenced by a multitude of factors, such as motivation, mood state (e.g., anxiety), fatigue, pain, etc. (Arnett, 2013). One of these secondary influences is the phenomenon of response expectancy: in response to particular stimuli, people may expect a specific (cognitive, emotional, physiological, etc.) effect, which then might actually occur as a consequence of the expectation (Kirsch, 1997).

Historically, the response expectancy effect has been examined in pharmacological and medical studies, through the placebo effect. The placebo effect refers to cases in which an individual expects an improvement in health or performance related to an inactive intervention that is described as beneficial, and experiences an actual beneficial effect associated with this positive expectation (Stewart-Williams & Podd, 2004). The placebo effect has its counterpart, called the nocebo effect, which refers to the negative effect resulting from negative expectations. The placebo-design paradigm permits one to examine the effect of a substance or a device, while controlling for expectancy effects. Additionally, in the field of psychology, the impact of response expectancy on cognitive performance has yielded intriguing results.

For example, Magalhães De Saldanha da Gama, Slama, Caspar, Gevers, and Cleeremans (2013) showed that the conflict resolution effect produced by the Stroop task can be moderated in response to a placebo suggestion wherein an inactive EEG cap is described as triggering a brain wave that influences color perception. Healthy participants who were told that the brain wave would impair their visual perception did indeed show more interference compared to baseline, while the opposite effect was observed for people told that the device would improve their visual capacities.

While some studies (Colagiuri, Livesey, & Harris, 2011; Magalhães De Saldanha da Gama et al., 2013) showed both nocebo and placebo effects on cognitive performance, other studies either observed an impact of only one of the two phenomena or investigated only one of them. For example, Oken et al. (2008) showed a placebo effect on memory, inhibition, and choice reaction time tasks for people receiving a pill that was supposed to improve their cognitive functioning (compared to the control group), but did not investigate the nocebo effect. In a study by Kvavilashvili and Ellis (1999), participants received an inactive substance presented as either a memory-enhancing pill (placebo group) or a memory-impairing pill (nocebo group). The results showed that, while participants in both groups perceived cognitive changes in the expected direction – an enhancement in the placebo group and an impairment in the nocebo group – a response expectancy effect on objective memory performance was observed only in the nocebo group: participants who were given the “memory-impairing” pill performed worse on memory tasks.

In sum, while some studies have shown a clear effect of both positive and negative expectancy manipulations on cognitive performance (e.g., Magalhães De Saldanha da Gama et al., 2013; Colagiuri et al., 2011), other studies have found conflicting results. One study demonstrated a placebo effect on cognitive self-assessment but not on objective cognitive tasks (Kvavilashvili & Ellis, 1999), another showed expectancy effects in some tasks but not in others (Oken et al., 2008), and one found nocebo effects but no placebo effects (Kvavilashvili & Ellis, 1999). These diverse findings do not call the existence of response expectancy effects themselves into question, but they do suggest that placebo and nocebo effects are complex phenomena emerging from a combination of situational and individual factors that can moderate the effect of the experimental placebo/nocebo instructions.

In this regard, some studies have tried to identify a placebo responder personality by examining different aspects of personality such as optimism (Geers, Helfer, Kosbab, Weiland, & Landry, 2005; Geers, Kosbab, Helfer, Weiland, & Wellman, 2007; Morton, Watson, El-Deredy, & Jones, 2009) or neuroticism (Darragh, Booth, & Consedine, 2014; Pecina et al., 2013). In these studies, the dependent variable consisted mostly of subjective reports of changes in emotional state, pain, comfort, etc. To our knowledge, no study has yet examined personality moderators of response expectancy on objective cognitive performance. In our study, we focused specifically on the personality dimension of self-transcendence (ST; Cloninger, 1999) which has received growing interest in different psychology fields for several years now (Garcia-Romeu, 2010). ST is defined as “the self-forgetful awareness that all objects are integral participants in the evolution of all that is as a whole, giving coherence and joy to all things” (Cloninger, 1999a, p. 177). Three characteristics define people who are high in ST: a tendency to be absorbed when doing activities (people appear “absent minded”); a feeling of strong connectedness with the universe (and with nature) and other people; a propensity to spirituality including religion, belief in extrasensory experiences or telepathy (see also MacDonald & Holland, 2002).

Interestingly, some studies showed that ST was linked to greater response to hypnotic suggestion. Cardeña and Terhune (2014) showed that participants who scored higher on ST reported higher perception of the announced changes suggested by the hypnotic suggestion (see also Laidlaw, Dwivedi, Naito, & Gruzelier, 2005). So, some studies have already shown that people high in ST could be especially susceptible to expectancies (in this case, expectancies induced by hypnotic suggestion). Moreover, the proposed role of self-transcendence in the expectancy effect on cognitive performance makes sense when one considers research by Hyland and collaborators (Hyland, Geraghty, Joy, & Turner, 2006; Hyland & Whalley, 2008; Hyland, Whalley, & Geraghty, 2007). These authors examined the link between the placebo effect on subjective symptoms (e.g. related to sleep quality) and spirituality, a personality trait that includes a dimension of transcendence. They found that this link depended on the type of therapy used (Hyland & Whalley, 2008; Hyland et al., 2007) and that spirituality, which involves two main dimensions – it “(a) includes a reference to transcendence or the sacred… and (b) emphasises an individual connection with transcendence, others, and the world in general,…” (Saroglou & Muñoz-García, 2008, p. 88) – was in some cases a dispositional predictor of placebo effects.

Regarding these results, ST could influence individuals’ response to placebo/nocebo instructions, and therefore moderate the expectancy effect. Indeed, high-ST individuals are prone to accept situations characterized by uncertainty and ambiguity and without any possibility of control; according to Cloninger (1999a), this is why they are often perceived as naïve. Because they are more likely to be absorbed in experience, to believe in supernatural, to feel connectedness with the environment, and to have a propensity for spirituality (Cloninger, 1999a; MacDonald & Holland, 2002), high-ST individuals could possibly be more prone to response expectancy effects than low-ST individuals. To our knowledge, no study has examined the moderating impact of ST on expectancy effects, nor – more generally – personality predictors of expectancy effects on cognitive performance, leaving the way open for further investigations.

Finally, another variable that could explain the unsystematic nature of the expectancy effect relates to the kinds of cognitive processes measured. Some cognitive functions may be more prone to produce expectancy effects than others. In this regard, the stereotype threat phenomenon (Steele & Aronson, 1995) reveals that reminding participants about a negative stereotype (e.g. “women are bad in math”) induces poor performance in targeted individuals. Because stereotype threat can be seen as a particular instance of the nocebo effect (i.e., reduction in performance that might be explained by negative expectancy effects induced by the activation of the stereotype), this literature could be helpful in understanding what kinds of cognitive processes are more susceptible to expectancy effects. Several authors have precisely shown that the negative expectancy induced by social representations is most likely to impact tasks that tap the most into executive processes, i.e. high-level cognitive processes which permit regulation during goal-directed actions (Croizet et al., 2004; Dardenne et al., 2013; Schmader & Johns, 2003; Schmader, Johns, & Forbes, 2008).

Consequently, it may be that nocebo conditions lead to underperformance in tasks that recruit mainly executive processes, while the placebo condition might lead to improvements in the same tasks. The results obtained by Oken et al. (2008) partially support this hypothesis as they observed a placebo effect on almost all tasks known to tap executive processes (delayed recall tasks, inhibition task, and multiple reaction time task), although there were no improvements in working memory and fluency tasks, which are also known to involve executive processes. In the expectancy effect literature, only a few studies (e.g., Looby & Earleywine, 2011; Oken et al., 2008; Sun, Zhang, He, Liu, & Miao, 2007) have examined the impact of experimental instructions on a set of cognitive tasks administered during the same session. In our view, it would be interesting to study the differential impact of expectancy effects on a series of cognitive tasks that differ according to the relative involvement of executive processes.

In line with this executive hypothesis, previous studies have also shown that placebo instructions lead subjects to rely more on controlled strategic processes. In these studies, it has been argued that the fact that participants spent more time reading misleading items in a memory task (Parker, Garry, Engle, Harper, & Clifasefi, 2008) and completing the primary task in a dual task condition (Parker, Garry, Einstein, & McDaniel, 2011) reflects the intervention of metacognitive processes. The allocation of more time to complete tasks is traditionally viewed as an indirect measure revealing the use of controlled strategic processes (Dunlosky & Metcalfe, 2009). However, this proxy measure does not allow us to be sure that metacognitive processes are being assessed in this way. To be certain of this, more direct measures of metacognition should be used. Traditionally, direct metacognitive processes are assessed by asking participants to judge their mental cognitive states while they carry out the task, an ability that is usually called metacognitive monitoring. During a memory task, a classic procedure for appraising these cognitive states consists in asking participants to judge the quality of their learning (judgement of learning; JOL) (Dunlosky & Metcalfe, 2009). In this context, there is reason to assume that response expectancy instructions would also affect the accuracy of participants’ metacognitive judgements, as assessed through a JOL procedure.

### Overview of the Present Study

In this study, our main goal was to investigate the expectancy effect (nocebo and placebo) on cognitive performance. More specifically, we aimed to study the moderating role of the self-transcendence dimension of personality on the placebo and nocebo effects on cognitive tasks. A second aim of this study was to examine which cognitive processes are impacted by the expectancy effect. To test this, participants were asked to complete cognitive baseline tasks to ensure for cognitive equivalence between groups, then to face three conditions (nocebo, neutral, and placebo) under which they completed attentional, memory, or executive tasks. Each participant undertook all tasks in random order and under randomized conditions (see below). Finally, participants completed a measure of perceived changes in cognitive functioning and a scale assessing ST. We hypothesized (1) that expectancy effects would be greatest for high-ST participants; (2) that tasks that call on executive processes (executive and memory tasks) would be preferentially negatively impacted by nocebo instructions and positively impacted by placebo instructions; (3) that placebo instructions would lead to better monitoring as evaluated by JOL, while nocebo instructions would lead to worse JOL accuracy, that is, inaccurate judgement of learning.

## Method

### Participants

Eighty healthy participants took part in this study. They were recruited on the campus of the University of Liège (Belgium). People were invited to participate in a study that allegedly investigated the impact of light on cognition. Potential participants were excluded if they reported a past or present neurological, developmental, or psychiatric disorder. Participants’ ages ranged from 18 to 30 years, with a mean age of 22.71 (*SD* = 3.11) years; years of education ranged from 9 to 21, with a mean education level of 13.44 (*SD* = 2.20) years; and 52.5% of the participants were female. Age and education level were controlled such that they were statistically equivalent (*ps* > .217) between experimental conditions (see below).

### Material

The information letter indicated that the university lab was carrying out multiple experiments on the effects of light on cognition. It was explained that human eyes accommodate to the illumination level; some levels of illumination have no effect on cognition (below 1000 lux), but at higher levels (ranging from 1000 to 7000 lux) light has a beneficial effect on cognition because rhodopsin and nerve impulses are optimally stimulated. It was also explained that, above this level of illumination (higher than 7000 lux), light has a depleting impact on cognition because rhodopsin, and therefore nerve impulses, are over-stimulated. This letter also informed participants that they would not be able to perceive the difference between lamps because the lamps impacted cognition by subtle chemical messages and nerve impulses. Of course, information about the lamps was totally misleading. Finally, in each case, participants were asked to keep the kind of lamp a secret from the examiner, supposedly so the examiner would remain unbiased.

To make the supposed experimental basis of the study appear more credible, we used three lamps with electric devices (e.g., a dimmer), and a label (patent for lamps) on their bases. In reality, these lamps had exactly the same electrical properties. Every lamp had the same bulb and an audible device that was briefly activated by the experimenter while switching on the lamp. Each lamp was accompanied by an information card that briefly described its anticipated effects. Information for the placebo (nocebo) lamp was: “You will be exposed to the lamp which improves (diminishes) and stimulates (inhibits) attentional and memory capacities. With this light, people feel a sensation of ease (being swamped) while completing memory and concentration tasks. People are more (less) alert, concentrate better (worse), find it easy (difficult) to memorize things, work faster (slower), and become tired less (more) quickly. Thus, exposure to this lamp will have a positive (negative) effect on your memory and your concentration. Don’t forget that you must not tell the experimenter the content of this information.” Information on the neutral lamp was exactly the same, except that it explained that the lamp had no effect on the various capacities mentioned.

#### Cognitive baseline tasks

These tasks, which differed from the tasks of interest (dependent variables), were administered prior to any expectancy instructions (i.e., without any lighted experimental lamp), to statistically control for prior inter-individual cognitive differences in analyses of expectancy effects.

*Computerized adaptation of the visual memory span subtest of the Wechsler Memory Scale – Revised (WMS-R; Wechsler, 1987)*. In this computerized task created with Toolbook software (version 11.0, SumTotal Systems Inc., Gainesville, FL), there are eight blue squares on the screen which turn red in a particular sequence. The participant’s task is to reproduce the opposite sequence by mouse-clicking on the squares. The length of the sequences increases until participants fail on three items of the same sequence length.

*Stroop task (Godefroy & GREFEX, 2008)*. This task, originally designed by Stroop (1935), is composed of three subtasks: a color naming subtask in which participants have to name the colors of rectangles as quickly as possible; a word reading subtask in which participants have to read color names as quickly as possible; and a color word naming subtask in which participants have to name the print color of color words (e.g., red written in blue) as quickly as possible, resisting the interference produced by the automatic tendency to read.

#### Experimental tasks

Participants completed three blocks (an executive block, a memory block, and an attention block), each comprising two tasks measuring either executive, memory or attentional functioning. Each block was administered following the experimental instructions and with the participant facing one of the three experimental lamps. Participants were exposed to each condition (lamps) and completed one of the three blocks under it. The order of the tasks within each block is presented below.

##### Executive block

We selected two tasks known to tax executive resources (i.e., a switching and a go/no-go paradigm) and to recruit primarily prefrontal areas (Collette & Van der Linden, 2002; Niendam et al., 2012). According to the executive hypothesis concerning the social negative expectancy effect, we expected these tasks to be affected by our experimental instructions.

*Go/No-go subtest of the Test of Attentional Performance (TAP; Zimmermann & Fimm, 2010)*. In this computerized inhibition task, two kinds of crosses (+ and X) rapidly appear on the screen one at a time. Participants have to press the response button as quickly as possible only when the X appears.

*Flexibility subtest of the TAP (Zimmermann & Fimm, 2010)*. In this computerized flexibility task, a letter and a digit appear simultaneously on the screen, with their position changing randomly (left or right side of the screen). Participants have to respond with one of two response buttons (left or right). They must alternate as quickly as possible between pressing the button corresponding to the location of the letter for one item, and then pressing the button corresponding to the location of the digit for the following item, and so on. Since a trend toward alternating between left and right hands becomes pronounced during the task, items requiring participants to press the same button twice (68 out of a total of 100 items) tax executive processes because they require the on-going (and therefore dominant) response of alternating between left and right hands to be inhibited.

##### Memory block

The following memory tasks are known to recruit not only memory processes but also executive resources. Delayed word recall involves executive functioning (Fletcher, Shallice, & Dolan, 1998), and the Self-Ordered-Pointing-Test (SOPT) is a working memory task calling on executive resources (Bryan & Luszcz, 2001). According to the literature on social negative expectations and previous expectancy effect studies showing the impact of placebo/nocebo instructions on memory performance, these tasks may be influenced by experimental conditions.

*Self-Ordered Pointing Test (SOPT; Petrides & Milner, 1982)*. In this computerized working memory task created in Toolbook 11.0, abstract symbols are displayed on the screen, and the participants’ task is to move the mouse over each symbol. But each time participants select a symbol, the location of all the symbols changes. Therefore, participants have to remember which symbols they have already selected. There are five series (4, 5, 6, 8, and 10 symbols) of two groups with the same symbols: the second group requires participants to begin with a different symbol than the first one and therefore recruits more executive processes (Geurten, Catale, & Meulemans, 2015a).

*Word pair memory task (Froger, Bouazzaoui, Isingrini, & Taconnat, 2012)*. This memory task created in E-Prime software 2.0 was adapted from the experimental task used by Froger et al. (2012). During the learning phase of our task, 30 word pairs are randomly displayed on the screen one by one for an indefinite period of time (until participants press the space bar), as the learning phase is self-paced. During this learning phase, after each word pair, participants are encouraged to state aloud a judgement of learning (JOL), indicating their confidence in their capacity to recall this word pair later, on a scale ranging from 0% to 100%. During the cued-recall phase (after a delay of approximately 10 minutes filled with a non-verbal cognitive task, the SOPT), the first word of each pair is individually displayed on the screen in random order, and the participant’s task is to recall the associated word.

##### Attention block

The following attentional tasks rely heavily on processing speed and less on controlled processes (Van Zomeren & Brouwer, 1994). We therefore expected that these tasks would not be influenced by expectancy effects.

*Shortened version of the vigilance subtest of the TAP (Zimmermann & Fimm, 2010)*. In this vigilance task created in E-Prime software (version 2.0, Psychology Software Tools, Inc., Sharpsburg, PA), two adjacent rectangles remain on the screen and become grey one at a time. Occasionally, though, this alternation sequence is broken; when that happens, participants have to press the space bar as quickly as possible.

*D2 test (Brickenkamp, 1998)*. In this paper-and-pencil processing speed task, participants have to cancel out “D2” signs (letter D with exactly two dashes located below and/or above it) dispersed among distractors (letter D with fewer or more than two dashes and letter P with any number of dashes). Participants have to process 14 lines of P’s and D’s, for which 20 seconds are allocated per line.

#### Questionnaires

*Perceived effect questionnaire*. This 6-item scale (Cronbach’s α = .88) was specifically created for this experiment but no validation studies were conducted. This scale requires participants to respond to statements regarding their perception of the effect of the lamps (e.g., “I do not think the lamps had the anticipated effect”) using a 5-point Likert scale ranging from “totally disagree” to “totally agree,” with a “neutral” midpoint. (The questionnaire is presented in Appendix A).

*Self-transcendence subscale of the Temperament and Character Inventory – Revised (TCI-R) (Cloninger, 1999b)*. This 26-item scale (Cronbach’s α = .84) evaluates the self-transcendence dimension of personality and is a subscale extracted from the TCI-R of Cloninger (1999b). The TCI-R has been developed from the psychobiological model of temperament and character (Cloninger, Svrakic, & Przybeck, 1993). This subscale assesses the three facets of ST described in the introduction section: the tendency to absorption (“creative self-forgetfulness”), the sense of strong connection with the universe as a whole (“transpersonal identification”), and the propensity to spirituality (“spiritual acceptance”). Participants respond on a 5-point Likert scale (ranging from “totally false” to “totally true”), indicating how well each statement describes them. The French version of the TCI-R has shown good psychometric characteristics (Hansenne, Delhez, & Cloninger, 2005).

#### Measures and data reduction

From both theoretical conceptualizations and simple bivariate correlations between scores on tests, we computed composite scores in order to reduce the number of dependent variables. We created a composite attention score by averaging the attention index of the D2 test (total number of correctly treated D2 items minus total number of errors) and the median reaction time of the vigilance task (reversed) (*r* = .26, *p* = .021). A composite memory score was created by averaging Z-scores for the number of correctly recalled words during the delayed recall of the word pair memory task and the total number of errors of the SOPT (reversed) (*r* = .31, *p* = .005). For the accuracy of JOL during the word pair memory task, gamma correlations were calculated for each subject’s JOL and their actual word recall. In this case, the higher the correlation, the more accurate the JOL. Regarding executive functions, since the total number of errors on the go/no-go subtest (“Inhibition score”), and the total number of correct responses (CR) for items without a hand change (“Flexibility score”) were not correlated (*r* = –.13, *p* = .267), we performed statistical analysis on each one separately.

For the cognitive baseline tasks, we selected the following measures: total number of errors for the color word naming subtest of the Stroop task (“Stroop interference”), completion time for the color naming subtask of the Stroop task (“Stroop color”), and total number of correctly answered items in the visual span subtest (“Span score”).

### Procedure

The experiment took place either in a university lab or at the participant’s home (63.8% of participants took part in this study at home).^1^ After participants gave their written consent, sociodemographic information was collected (age and years of education), and participants completed the two cognitive baseline tasks. Then, they read a letter informing them that the goal of the study was to test the effect of three experimental lamps that had differential effects on cognition (positive, negative, or no effect). Next, each participant was exposed to each lamp – all of which were actually identical – while completing the tasks. There were three blocks, each comprising two tasks: executive tasks, attentional tasks, and memory tasks. Each block was performed with a different lamp, with the order of lamps and blocks being totally randomized (e.g., participant A completed the executive block thirdly facing the nocebo lamp while participant B completed the executive block first, and facing the neutral lamp). As such, this between-subject design was created following the Latin square procedure. Before presenting each block of tasks, the experimenter switched off the room light and switched on the experimental lamp. Each experimental lamp was accompanied by an envelope containing information regarding the lamp’s effect, which participants were invited to read silently. Thus, the experimenter remained blind to the condition. Between lamp conditions, the experimenter switched on the room light, telling participants that their retinas had to readapt to the room light before being exposed to the next lamp. After completing all task blocks, participants filled in a perceived effect questionnaire and the self-transcendence subscale of the TCI-R (Cloninger, 1999b). Finally, they were debriefed on the real purpose of the study.

## Results

Statistical analyses were performed using SPSS.

### Perceived Effect of Lamps

We created an index of perceived effect by averaging the 6 items of the scale. Higher scores represent a tendency to feel the anticipated effects of the different lamps. Descriptive statistics showed that 41% of participants obtained a mean score between 1 and 3, inclusively (*M* = 2.35, *SD* = 0.52), suggesting that they did not feel the effects of the lamps. However, 59% of participants obtained a score above 3 (*M* = 3.67, *SD* = 0.30), indicating that they felt the lamps’ effects. Interestingly, there was a positive correlation between perceived effect of lamps and ST (*r* = .25, *p* = .025).

### Moderation Analyses

Since we were interested in understanding for which participants expectancy effects impact cognitive performance, we chose to conduct moderation analyses. The goal of moderation analyses is to examine whether the size or the sign of the effect of one variable (X; expectancy instructions) on another (Y; cognitive score) is conditioned by (or depends on) one or more other variables (M; ST). To test our hypothesis regarding the moderating role of ST, we followed the regression-based approach recommended by Hayes (2013). This approach enabled us to study the conditional effects (i.e., the effect of X on Y depending on M) of experimental conditions on each dependent variable while keeping the ST moderator under the continuous metric (rather than dichotomizing ST). In other words, it enabled us to examine if there was an impact of expectancy instructions for all individuals or only for high-ST participants. We therefore expected an interaction between expectancy instructions and ST. To do so, we used Hayes’ (2013) PROCESS computational tools.

Due to our multi-categorical independent variable,^2^ we used orthogonal contrasts to test the nocebo effect and the placebo effect separately in regression analyses (Brauer & McClelland, 2005; Davis, 2010; Judd, McClelland, Ryan, Muller, & Yzerbyt, 2010). On the one hand, when examining the nocebo effect, the independent variable was the nocebo contrast (the nocebo condition contrast-coded +1/2 and the neutral condition contrast-coded –1/2) and we controlled for (as covariate) the associated orthogonal contrast (the nocebo and the neutral condition contrast-coded –2/3 and the placebo condition +2/3) and its interaction with ST. On the other hand, when analyzing the placebo effect, the independent variable was the Placebo contrast (the placebo condition contrast-coded +1/2 and the neutral condition contrast-coded –1/2) and we controlled for the associated orthogonal contrast (the placebo and the neutral conditions contrast-coded –2/3 and the nocebo condition +2/3) and its interaction with ST. By doing this, we conserved the same sum of squares. Consequently, for each dependent variable, we conducted an ordinary least squares regression in which the cognitive score was estimated from the nocebo or placebo contrast, ST, and their interaction, while controlling for the cognitive baseline performance and the associated orthogonal contrast (as well as its interaction with ST). In these analyses, ST was mean-centered. Therefore, the *b* coefficient for experimental condition, for instance, indicates the conditional effect of condition when ST was at its mean value. Finally, regarding cognitive baseline performance, we controlled for Stroop interference when analyzing executive tasks; Stroop color for the attentional score; and Span score for the memory score and JOL accuracy.

#### Executive tasks

##### Flexibility score

There was no significant effect of the Stroop interference covariate (*p* = .892). There was a significant effect of ST (*b* = –1.99, *SE* = .76, *t* = –2.62, *p* = .011). Regarding the placebo condition, there was no effect of placebo contrast or interaction between placebo contrast and ST (*ps* > .493). However, at the mean value of ST, there was a significant effect of nocebo contrast (*b* = –1.96, *SE* = .94, *t* = –2.09, *p* = .040). More interestingly, as predicted, the interaction between nocebo contrast and ST was significant (*b* = –5.04, *SE* = 2.02, *t* = –2.50, *p* = .015). To probe this interaction, we used the pick-a-point approach, which creates two values of the ST score: low ST (one standard deviation below the mean) and high ST (one standard deviation above the mean). The results showed that there was a nocebo effect only for high (*b* = –4.58, *SE* = 1.44, *t* = –3.18, *p* = .002) ST individuals, who produced fewer correct responses than their counterparts in the neutral condition. There was no effect of condition for low-ST participants (*p* = .626).

##### Inhibition score

There was no significant effect or interaction on the inhibition score (*ps* > .175).

#### Attention tasks

##### Composite attention score

Apart from a significant effect of the Stroop color covariate (*b* = –.04, *SE* = .01, *t* = –4.32, *p* < .000) on the Attention score, there were no other significant effects or interactions (*ps* > .119).

#### Memory tasks

##### Composite memory score

There was a significant effect of the Span score covariate (*b* = .13, *SE* = .04, *t* = 3.19, *p* = .002) on the memory score, but no effect of ST or placebo contrast, or interaction between placebo contrast and ST (*ps* > .244). As well, at the mean value of ST, there was no effect of nocebo contrast (*p* = .106). However, there was a trend toward an interaction between nocebo contrast and ST (*b* = .73, *SE* = .38, *t* = 1.95, *p* = .055). When this interaction was probed, the results showed that there was an effect of the nocebo instructions only for high-ST participants (*b* = .72, *SE* = .28, *t* = 2.55, *p* = .013), who performed *better* than their counterparts in the neutral condition. There was no effect of nocebo condition for low-ST participants (*p* = .888).

##### Accuracy of JOL

There was a significant effect of the Span score covariate (*b* = –.06, *SE* = .02, *t* = –3.99, *p* < .000), but no effect of ST (*p* = .131) and nocebo contrast (*p* = .242). As well, there was no significant interaction between nocebo contrast and ST (*p* = .872). However, there was a significant effect of placebo contrast (*b* = .19, *SE* = .08, *t* = 2.34, *p* = .022), indicating better JOL accuracy in the placebo condition than in the neutral one at the mean value of ST. The three-way interaction was not significant (*b* = .04, *SE* = .16, *t* = .27, *p* = .789). However, we carried out specific contrasts to test our hypothesis that a placebo effect would be found only for high-ST participants. Simple effects showed that there was a placebo effect for high-ST (*b* = .21, *SE* = .11, *t* = 1.85, *p* = .069) participants, but not for low-ST participants (*p* = .156).

In sum, the results indicated no effect of expectancy instructions on the attention score or on the inhibition task. Regarding the flexibility score (executive task), no placebo effect was found. However, we observed a decrease of performance for moderate- and high-ST participants in the nocebo condition compared to the neutral one. Regarding the memory score, there was no effect of placebo instructions, but an unexpected effect of nocebo instructions for high-ST participants: they scored better in the nocebo condition compared to the neutral condition. Finally, there was no effect of nocebo instructions on the JOL accuracy, but a placebo effect for moderate- and high-ST participants: they scored better following placebo instructions compared to the neutral ones.

## Discussion

As hypothesized, the ST personality dimension moderated participants’ responses to experimental instructions, with only moderate- and high-ST participants exhibiting improved or decreased performance following experimental instructions. As expected, our experimental instructions had no impact on attentional tasks but did have an impact on executive and memory tasks. Indeed, nocebo instructions led (moderate- and) high-ST participants to perform worse on the flexibility task but, unexpectedly, better on memory tasks. Furthermore, we found that placebo instructions led to more accurate judgements of learning, compared to the neutral condition.

### Influence of ST

As predicted, ST moderated the impact of the expectancy effect on cognitive performance. Nocebo instructions had an effect on the flexibility (impaired performance) and memory (improved performance) scores and a placebo effect on JOL but only for moderate- and high-ST participants. Concerning the moderating role of ST, as expected, the higher participants’ ST was, the worse they performed on a flexibility task following the nocebo instructions and the better they judged their learning following placebo instructions. Unexpectedly, high-ST participants got better memory scores in the nocebo condition than in the neutral condition. Finally, the higher participants were on ST, the more they reported having felt the expected changes facing the lamps. Our results are consequently in accordance with the results of Cardeña and Terhune (2014) who showed that higher ST is associated with higher experiential hypnotic suggestibility. As well, our results are in accordance with Hyland and Whalley (2008) who showed that in a spiritual context, spirituality – which includes ST dimension – moderates the expectancy effect on subjective reports. These results and our findings could imply that a strong feeling of ST predisposes individuals to expectancy effects.

People high in ST are characterized by three facets (Cloninger, 1999a). The creative self-forgetfulness facet describes high ST individuals as having a tendency to absorption. Absorption – defined as a “*disposition for having episodes of “total” attention that fully engage one’s representational (i.e., perceptual, enactive, imaginative, and ideational) resources*” (Tellegen & Atkinson, 1974, p. 268) – has been found to be positively linked to both hypnotic suggestibility and self-transcendence (Cardeña & Terhune, 2014). Then, in our study, participants high in ST could have been especially susceptible to expectancy effects because they did not question the purpose of the experiment and were truly committed to it. Secondly, the transpersonal identification facet describes high ST individuals as feeling deep union with the Universe, including the physical environment. Because people high in ST perceive themselves as a part of a whole, they could have been especially receptive to the idea that the environment (i.e. the lamps) could affect them and modify their experiences. The third facet – spiritual acceptance – characterizes individuals high in ST as believing in extrasensory experiences as well as other spiritual influences like telepathy. And, spirituality has been found in some cases to predispose individuals to placebo effects (Hyland & Whalley, 2008). Their willingness to accept and believe in spiritual or extrasensory experiences could also have led high-ST participants to be especially swayed by our experimental instructions. Due to the present sample size, we could not afford to study specifically these different dimensions. Subsequent studies should therefore focus on the specific facets of self-transcendence to better understand its role in the expectancy effect.

### An Executive vs. Difficulty-based Account of the Nocebo effect

According to several authors (e.g., Schmader et al., 2008), the social negative expectations effect, which is a specific case of the nocebo effect, preferentially impacts executive functions. Our results are partially consistent with this hypothesis. Indeed, there was no effect on tasks that minimally tap executive processes (attentional tasks), but there was an effect on the flexibility task and an effect on memory tasks, although in the unexpected direction. However, nocebo instructions had no effect on the inhibition task. In fact, the inhibition task appears to be too easy: participants made a mean of 0.65 errors, and 48 out of the 80 participants made no errors at all. While it does recruit executive resources, this task may not have been sensitive (floor effect) for our participants, who were composed principally of university students (no inter-individual variability).

While, as expected, the nocebo instructions had a deleterious impact on the flexibility score, they were associated with an improvement in memory performance. These two tasks, however, are not equally difficult. As a matter of fact, several studies in the stereotype threat literature have shown that task difficulty strongly moderates the impact of negative expectancy (e.g., Neuville & Croizet, 2007; O’Brien & Crandall, 2003). In these studies, following stereotype threat instructions, participants’ performance was found to decrease in complex tasks, while it improved in simpler tasks. This difficulty-based account could explain the differential effect of the nocebo instructions on the flexibility task (poorer performance) and the memory tasks (better performance). Indeed, the memory tasks were self-paced while the flexibility task involves time pressure. Since there was no time limit during the memory tasks, high-ST participants – who were committed to the experiment – may have improved their performance following nocebo instructions. For example, they may have been more cautious (taking more time) due to the nocebo instructions. Consequently, they produced fewer errors. This strategy could not be applied in the flexibility task because of the time pressure.

To sum up, nocebo effects on neuropsychological tasks may have been determined not only by the kinds of cognitive processes involved (executive vs. non-executive), but also by the complexity of the tasks. However, this explanation should be investigated more systematically, for example by varying the level of difficulty (simple vs. complex) within the same task (executive vs. non-executive). Furthermore, we think that not only objective difficulty but also – in fact, primarily – perceived difficulty plays a major role in producing the expectancy effect (e.g., Schuster, Martiny, & Schmader, 2015). If individuals find a task difficult in a nocebo condition, this perceived difficulty may reinforce the experiment’s credibility and therefore the nocebo effect, in a vicious circle.

### Placebo Effect: Impact on Metacognitive Processes, but not on Cognitive Performance

Using indirect measures of monitoring, Parker et al. (2011) and Parker et al. (2008) showed that placebo instructions lead to better metacognitive processes. A secondary goal of our study was to investigate the expectancy effect on a direct measure of monitoring, namely judgement of learning. Our results showed that there was a placebo effect for moderate- and high-ST participants; that is, they assessed their own performance on the task more accurately than their counterparts in the neutral condition. Therefore, inducing positive expectancies led participants to better assess their cognitive state while completing a memory task. Our results suggest that placebo conditions lead to better monitoring strategies. To our knowledge, this is the first study to show a placebo effect on a direct measurement of monitoring.

Although participants assessed their memory performance better in the placebo condition, placebo instructions did not lead to improvement in memory performance or executive tasks. This kind of result has been observed elsewhere. For example, Looby and Earleywine (2011) showed that placebo instructions impact subjective assessment but have no effect on objective performance. As well, Kvavilashvili and Ellis (1999) observed a nocebo effect on objective and subjective assessment of memory, while their placebo instructions only affected the subjective assessment of memory (not objective performance). Therefore, in the cognitive domain, the placebo effect appears not to systematically mirror the nocebo effect and to preferentially trigger changes in subjective assessment.

Another explanation could relate to the fact that we selected a lamp as our response expectancy object in order to use an object about which participants would have no a priori expectations regarding its effect on cognition. However, response magnitude could have differed between the nocebo and placebo effects (limited to subjective assessment) because the cognitive-enhancing characteristic of light (placebo instructions) was perceived as less powerful than the cognitive-impairing instructions. Unfortunately, our design prevented us from disentangling the perceived effects of the placebo lamp from those of the nocebo lamp.

## Conclusions

Our study showed that nocebo instructions lead to a decrease in flexibility and an increase in memory performance. To the best of our knowledge, this is the first study to show an effect of placebo instructions on a direct measure of cognitive control, namely the accuracy of judgements of learning. These response expectancy effects appeared to be moderated by self-transcendence. This study also confirms the very complex nature of the mechanisms involved in expectancy effects, suggesting that some specific conditions are required for the effects to occur. Our study is a first step in identifying and disentangling the factors that might influence the appearance of placebo/nocebo effects on cognitive functioning, as well as the expectancy effect on higher-order cognitive functions (i.e., metacognitive processes). As such, our study may also enrich the literature on stereotype and benevolent threats in helping to understand which specific cognitive functions these threats can impair.

## Additional File

The additional file for this article can be found as follows:

10.5334/pb.364.s1 Appendix Perceived effect questionnaire.

## Footnotes

In cases where participants took part in the study at home, the experimenter brought the material (including lamps) with her. The experiment was conducted in a silent and isolated room of the participants’ house. Because some authors suggest that the location of the experiment can influence expectancy effects (Parker et al., 2008), we were interested in its moderating role. However, too few participants agreed to take part in the study at the lab (due to geographical constraints). We therefore omitted this variable in order to avoid type I errors due to minimal effective size in some cells (self-transcendence X lab X condition). Nevertheless, we verified that this variable did not influence statistical results by entering it as a covariate. Since the pattern of results stayed exactly the same, we disregarded this variable in the results section of this experiment.

In regression analyses, a categorical predictor with more than two levels should not be entered as a metric variable (in this study, this would lead to: Nocebo coded –1; Neutral coded 0; and Placebo coded +1). This would consist of applying an arbitrary linear relationship between levels of a non-ordinal variable (see Judd et al. 2010). This is why we use orthogonal contrast in this study. These orthogonal contrasts permit to directly test for group mean differences. Contrary to the omnibus test with several degrees of freedom, it then permits to test specific hypotheses (see Brauer & McClelland, 2005).
