# Supplementary material for: The Role of Self-Transcendence and Cognitive Processes in the Response Expectancy Effect
Source: Psychol Belg. 2017 Jun 26;57(2):77–92. doi: 10.5334/pb.364 (PMC6194523; doi:10.5334/pb.364)
Supplement: Appendix — Perceived effect questionnaire. [file pb-57-2-364-s1.pdf]

Appendix

Perceived effect questionnaire

1. Overall, I felt the expected effect of the lamps (e.g., greater ease with the 5000-lux lamp, which stimulates cognitive functioning).

Totally disagree Disagree Neutral Agree Totally agree

2. I do not think that the lamps had the anticipated effect.

Totally disagree Disagree Neutral Agree Totally agree

3. I felt the anticipated effect of the lamps on my concentration capacities and my ability to pay attention to tasks.

Totally disagree Disagree Neutral Agree Totally agree

4. I felt the anticipated effect of the lamps on my response speed.

Totally disagree Disagree Neutral Agree Totally agree

5. I felt the anticipated effect of the lamps on my memory capacities.

Totally disagree Disagree Neutral Agree Totally agree

6. I felt the anticipated effect of the lamps on my ability to perform tasks.

Totally disagree Disagree Neutral Agree Totally agree
